# Perivascular interactions and tissue properties modulate directional glymphatic transport in the brain

**DOI:** 10.1186/s12987-025-00668-3

**Published:** 2025-06-23

**Authors:** Chenji Li, Sadegh Dabiri, Arezoo M. Ardekani

**Affiliations:** 1 School of Mechanical Engineering, Purdue University, West Lafayette, IN 47906 USA; 2 Department of Agricultural and Biological Engineering, Purdue University, West Lafayette, IN 47906 USA

**Keywords:** Glymphatic system, Perivascular interactions, Brain fluid dynamics

## Abstract

The glymphatic theory suggests a convective transport mechanism through brain tissue, which has significant implications for both brain waste clearance and drug delivery. However, the existence and driving mechanisms of directional convection from periarterial to perivenous spaces remain debated. Additionally, the role of brain tissue stiffness in parenchymal transport remains unclear, as experiments have reported varying trends in stiffness changes in cases of aging and neurodegenerative diseases. Previous mechanistic models often simplify or neglect perivenous spaces and venous deformation, raising questions about whether arterial vasomotion alone can effectively drive artery-to-vein transport. In this study, we propose a multiphysics model that incorporates the poroelastic nature of brain tissue, capturing the dynamic interactions between periarterial and perivenous spaces. Our results demonstrate that net glymphatic flow sweeps from periarterial space across parenchyma and is modulated by the periarterial-perivenous interactions, leading to higher pressure in periarterial space that drives unidirectional bulk transport from periarterial space to perivenous space. We also show that brain tissue stiffness presents a non-monotonic effect on both the glymphatic transport and its efficiency, with their respective peaks occurring at different stiffness values. Notably, the glymphatic convection rate peaks at physiologically relevant levels of brain stiffness. Furthermore, phase-delayed venous vasomotion is found to enhance glymphatic flow. These findings highlight the critical role of perivascular interactions and provide a framework for exploring brain fluid dynamics and potential therapeutic strategies for neurodegenerative diseases.

**Supplementary Information:**

The online version contains supplementary material available at 10.1186/s12987-025-00668-3.

## Introduction

The transport mechanisms within the brain are crucial for understanding and managing central nervous system (CNS) disorders, such as Alzheimer’s Disease (AD), where the accumulation of biomarkers like amyloid-beta is closely linked to disease progression [19]. Effective therapeutic strategies, such as intrathecal (IT) injection, also depend on the understanding of the path of drug delivery to the target tissue for the prediction and optimization of drug design and treatment methodology [30, 39]. The glymphatic hypothesis, introduced as a mechanism for metabolic waste clearance, suggests that convective transport occurs within the brain [22]. It has been proposed that there is a flow through periarterial spaces (between arteries and glial layers), permeating the brain tissue, and exiting via the perivenous spaces (between veins and glial layers) [26, 31]. This directional convection from periarterial to perivenous spaces has gained support from both analytical and experimental evidence [12, 20, 22, 26, 40]. However, the efficiency and dynamical mechanism of this directional transport remain subjects of ongoing debate [20, 32].

A prevailing hypothesis suggests that peristaltic pumping, driven by pulsations in the blood vessel walls, causes a deformation wave that propels fluid flow along the perivascular space (PVS) and through the parenchyma [9, 18, 43, 51, 55]. This model aligns with observations of a strong correlation between cerebrospinal fluid (CSF) flow rate within PVS and the cardiac rhythms [33]. Despite its intuitive appeal, modeling efforts face several unresolved challenges that question this mechanism under physiological conditions. First, the relationship between brain tissue stiffness and glymphatic transport remains unclear. Some experimental studies have suggested that brain tissue softens with age [1, 44, 48] and in AD [11, 37], potentially correlating with reduced glymphatic clearance [27, 41]. Several models that couple poroelasticicy with periarterial dynamics have provided valuable insights into this trend [14, 43]. However, other experimental studies have observed brain tissue stiffening with age and AD [15, 17, 46]. Additionally, brain tissue softens during sleep compared to wakefulness [15, 16], which has been associated with enhanced glymphatic clearance during sleep [54]. These differing trends highlight the need for further investigation into the role of brain tissue stiffness on glymphatic transport. Second, the role of perivenous space complicates the understanding of glymphatic transport. The glymphatic hypothesis suggests a directional flow from periarterial to perivenous spaces [22]. In the flow resistance study [45] and network modeling [42, 49], the perivenous spaces are often considered as sink, cooperating with the source effect of periarterial spaces. However, many mechanistic models focus only on periarterial spaces or simplify perivenous spaces as regions of constant pressure [4, 14, 24, 51]. Given that veins are compliant and are subject to blood pressure fluctuations, it is reasonable to expect that venous deformation may influence glymphatic flow dynamics [20]. It remains unclear whether perivenous motions enhance or offset the flow generated by arterial pulsations. Moreover, studies suggest that peristaltic pumping by artery alone may be insufficient to drive efficient net flow under normal physiological conditions [23]. When neglecting pressure variation in perivenous spaces, oscillatory pressure in periarterial space would produce primarily back-and-forth convection with minimal net flow [14]. This observation highlights the need to consider the dynamic interaction between periarterial and perivenous spaces and parenchyma.

To help address these challenges, we propose a multiphysics model that captures the dynamic interaction between periarterial and perivenous spaces mediated through the poroelastic properties of brain tissue. Our findings indicate a net glymphatic transport originating from periarterial spaces and sweeping through parenchyma, modulated by the interaction between periarterial and perivenous spaces. The interaction between PVSs causes the pressure in periarterial spaces to consistently remain higher than in perivenous spaces, driving unidirectional bulk flow from periarterial to perivenous space. The brain tissue stiffness is found to play an important role, where both net glymphatic transport and efficiency show nonmonotonic responses to stiffness, with their respective peaks occurring at different stiffness values. Furthermore, we find that phase-delayed venous vasomotion enhances glymphatic transport, showing the importance of considering interactive periarterial and perivenous dynamics in understanding brain fluid dynamics. This study provides new insights into the mechanisms underlying directional glymphatic transport and emphasizes the critical role of perivascular interactions. Our model offers a framework to explore how vascular dynamics and tissue properties may affect waste clearance, with potential implications in neurodegenerative diseases and therapeutic strategies.

## Materials and methods

### Overview of the modeling framework

**Fig. 1:**
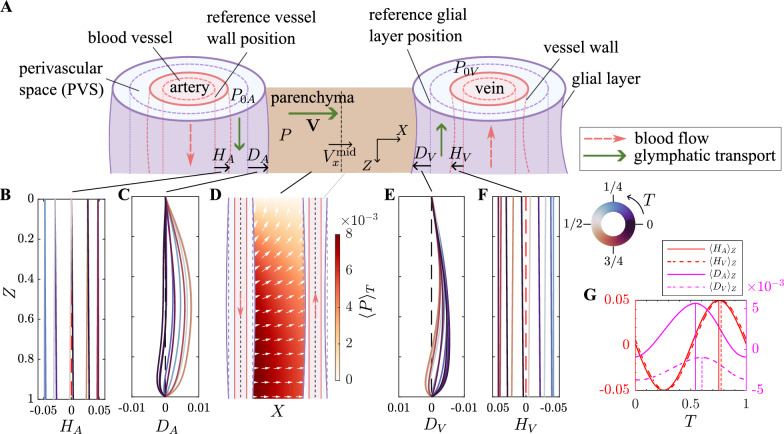
Representations of perivascular and parenchymal dynamics and the associated directional transport in the glymphatic system. **A** Schematic of arterial and venous perivascular spaces (PVSs) along with parenchymal and glial layers. $H_A$ and $H_V$ represent the radial deformation of artery and vein, respectively, with $D_A$ and $D_V$ denoting the radial deformation of glial layers. The transport direction is indicated for both blood flow and glymphatic transport. **B, C, E, F** Stroboscopic view of periarterial and perivenous spaces’ deformation over time, showing the oscillatory behavior of $H_A$, $H_V$, $D_A$, and $D_V$. **D** Time-averaged pressure and velocity fields within the parenchyma. The color shows the distribution of pressure, while the arrows indicate the direction and magnitude of velocity across the parenchyma. **G** Temporal evolution of spatially averaged deformation of PVSs over one oscillatory cycle.

The transport dynamics of glymphatic system involve CSF transport within the PVS, interstitial fluid (ISF) transport within the parenchyma, and their exchange through glial layers. We idealize the periarterial and perivenous spaces as parallel, axisymmetric cylindrical conduits [52], as illustrated in Fig. 1. Each cylinder has a length of *l*, bounded by the impermeable blood vessel wall with a neutral radius $r_1$ and the permeable glial layer with a neutral radius $r_2 = r_1 + b$, where *b* represents the neutral width of the PVS. The poroelastic parenchyma in between has width $l_p$, characterizing the average distance between the artery and vein. Further details on parameter selection are provided in the Sect. “*Parameter selection*”.

The PVSs are open channels that are filled with CSF and connected to the subarachnoid space (SAS) [26, 36]. The CSF flow within the PVS is governed by the Navier-Stokes equations. The inlet of PVS corresponds to the point where the penetrating arteries originate at the brain’s surface, sharing the same pressure with the SAS. The blind-ended outlet of the PVS is located deep within the brain parenchyma, associated with the transition point from the periarterial and perivenous spaces to the capillaries. Along the blood flow direction, the radial deformation wave of the vessel wall, *h*, propagates through the artery towards the deep parenchyma and then propagates through the vein towards the surface of the brain, aligning with the blood flow directions. The deformation wave is characterized by a sinusoidal wave of frequency *f*, amplitude $\bar{h}$, and wavelength $\lambda _w$. The deformation of the glial layer, denoted by *d*, modulates the dynamics of both PVS and parenchyma, while the flux *q* denotes the flow across the glial layer.

Biot’s theory is utilized to describe the poroelasticity of brain parenchyma (within the 2D domain marked as $\Omega$), where the living brain tissue is assumed to be fully saturated with ISF. As shown schematically in Fig. 1, the *z*-direction aligns with the PVS, pointing from the surface of the parenchyma toward deeper regions, and the *x*-direction points from the periarterial space to the perivenous space. At the interface with the PVS, the deformation, flow velocity, and hydrostatic pressure are continuous across the glial layer, and the permeable boundary on the brain surface facilitates the direct CSF-ISF exchange between brain tissue and the SAS. The brain tissue is characterized by Young’s modulus *E* and hydraulic permeability *k*.

To generalize the model, we nondimensionalize the length by *l*, radial deformation by *b*, time by 1/*f*, stress by the Lamé’s second parameter of tissue $\mu$, and pressure by $\eta f / \varepsilon ^2$, where $\eta$ is the dynamic viscosity of CSF, and $\varepsilon = b/l$ is the slenderness of the PVS. The pressure scale is chosen by assuming that the flow is primarily driven by the axial pressure gradient along the slender PVS, balancing the viscous resistance arising from radial velocity gradients. As CSF has low Reynolds number flow (where the Reynolds number characterizes the ratio of inertial to viscous forces) within slender PVS ($\varepsilon \ll 1$), the governing equation of CSF flow can be simplified using lubrication theory [14, 43]. In this study, we adopt a convention where lowercase letters represent dimensional variables, and uppercase letters represent dimensionless variables. The key dimensionless parameters are: (1) the dimensionless tissue stiffness $\mu ^* = \mu \varepsilon ^2 / (\eta f)$ that compares the elastic force to viscous force in brain parenchyma; (2) the dimensionless tissue permeability $K = k / b^2$ that compares the viscous forces between the parenchyma and the PVS; (3) the dimensionless amplitude of vessel radial deformation $\bar{H}$; and (4) the dimensionless wavelength of vessel deformation $\Lambda _w$. See “*Parameter selection*” for the selection of dimensional and dimensionless parameters, as well as their ranges used in the study.

### Governing equations of the PVS

The CSF flow within both periarterial and perivenous spaces is governed by the Navier-Stokes equations. Assuming axisymmetry and negligible variations in the tangential direction, the governing equations simplify to

1 $$\begin{aligned} & \frac{1}{r} \frac{\partial (r v_r)}{\partial r} + \frac{\partial v_z}{\partial z} = 0, \end{aligned}$$

2 $$\begin{aligned} & \rho \left( \frac{\partial v_r}{\partial t} + v_r \frac{\partial v_r}{\partial r} + v_z \frac{\partial v_r}{\partial z} \right) = - \frac{\partial p}{\partial r} + \eta \left( \frac{1}{r} \frac{\partial v_r}{\partial r} + \frac{\partial ^2 v_r}{\partial r^2} - \frac{v_r}{r^2} + \frac{\partial ^2 v_r}{\partial z^2} \right) , \end{aligned}$$

3 $$\begin{aligned} & \rho \left( \frac{\partial v_z}{\partial t} + v_r \frac{\partial v_z}{\partial r} + v_z \frac{\partial v_z}{\partial z} \right) = - \frac{\partial p}{\partial z} + \eta \left( \frac{1}{r} \frac{\partial v_z}{\partial r} + \frac{\partial ^2 v_z}{\partial r^2} + \frac{\partial ^2 v_z}{\partial z^2} \right) , \end{aligned}$$

where $v_r$ and $v_z$ are the radial (*r*) and axial (*z*) components of velocity $\textbf{v}$, *p* is the pressure, $\rho$ is the density of CSF, and $\eta$ is the dynamic viscosity of CSF. Boundary conditions are applied based on the physical characteristics of the PVS. At the inlet ($z = 0$) connected to the SAS, the pressure is set to equal to the reference SAS pressure $p \vert _{z = 0} = p_{\text {SAS}} = 0$. At the blind end of the PVS ($z = l$), the axial velocity is zero $v_z \vert _{z = l} = 0$. The vessel wall ($r(z, t) = r_1 + h(z, t)$) and the glial layer ($r(z, t) = r_1 + b + d(z, t)$) are deformable boundaries. The flow velocity on these boundaries accounts for the wall deformation and fluid exchange

4 $$\begin{aligned} v_r \,\vert _{r = r_1 + h}= & \frac{\partial h}{\partial t}, \quad v_z \,\vert _{r = r_1 + h} = 0. \end{aligned}$$

5 $$\begin{aligned} v_r \,\vert _{r = r_1 + b + d}= & \frac{\partial d}{\partial t} + q, \quad v_z \,\vert _{r = r_1 + b + d} = 0, \end{aligned}$$

where *h* and *d* represent the radial deformations of the vessel wall and glial layer, respectively, and *q* denotes the radial fluid flux across the glial layer. Due to the slender geometry of the PVS ($\varepsilon \ll 1$) and under the lubrication approximation, the normal vector of the glial layer is approximately aligned with the radial direction. Therefore, we consider the flux *q* to be primarily radial. To distinguish the arterial and venous PVS expressions, we use the subscripts $\cdot _A$ and $\cdot _V$ to denote the variables associated with the artery and vein, respectively. While in vivo measurements indicate that artery wall motion can be asymmetric [33], both sinusoidal [9, 25, 43] and asymmetric [23, 55] waveforms have been employed in previous computational studies. Because venous waveforms remain poorly known, introducing an asymmetric arterial waveform alone could create an artificial imbalance between periarterial and perivenous spaces and obscure our main focus on perivascular interactions. We adopt a sinusoidal model for both arterial and venous walls that captures the primary oscillatory and propagative features of vasomotion. Future studies can build upon this framework by incorporating asymmetric waveforms, particularly as more complete in vivo measurements of venous deformation—and ideally both arterial and venous waveforms in the same setup—become available. The arterial wall wave ($h_A$) propagates in the $+z$ direction

6 $$\begin{aligned} h_A (z, t) = \bar{h}_A \text{sin}\left[ 2 \pi z / \lambda _w - 2 \pi ft \right] , \end{aligned}$$

and the venous wall wave ($h_V$) propagates in $-z$ direction with a phase difference $\psi _{AV}$

7 $$\begin{aligned} h_V (z, t) = \bar{h}_V \text{sin}\left[ 2 \pi (2l - z)/ \lambda _w - 2 \pi ft + \psi _{AV}\right] , \end{aligned}$$

where $\bar{h}_A$ and $\bar{h}_V$ are the amplitude of vessel wall deformation on the artery and vein, respectively. The phase difference $\psi _{AV}$ is introduced to account for the phase delay due to the blood flow through capillary between arteries and veins. In our results, we observe that even a small phase difference $\psi _{AV}$ can enhance the glymphatic transport.

To nondimensionalize the governing equations, each dimensional variable (lowercase) is expressed in terms of its dimensionless counterpart (uppercase) and its characteristic scale

8 $$\begin{aligned} {\begin{matrix} & r = bR, \quad z = lZ, \quad t = \frac{T}{f}, \quad v_r = bf V_r, \quad v_z = lf V_z, \quad p = \frac{\eta f}{\varepsilon ^2} P, \quad \\ & q = bfQ, \quad r_1 = b R_1, \quad h = b H, \quad d = b D, \quad \lambda _w = l \Lambda _w. \end{matrix}} \end{aligned}$$

Based on the lubrication theory [14, 43], valid for $\varepsilon \ll 1$ and low Reynolds number flows, the leading-order dimensionless pressure $P_0$ satisfies

9 $$\begin{aligned} C_6 \frac{\partial ^2 P_0}{\partial Z^2} + C_7 \frac{\partial P_0}{\partial Z} = \frac{\partial H}{\partial T} (R_1 + H) - \left( \frac{\partial D}{\partial T} + Q\right) (R_1 + 1 + D) \end{aligned}$$

with boundary conditions

10 $$\begin{aligned} P_0 \,\vert _{Z=0} = 0, \quad \frac{\partial P_0}{\partial Z} \,\vert _{Z=1} = 0. \end{aligned}$$

The coefficients $C_6$ and $C_7$ are functions of *H* and *D*, defined as

11 $$\begin{aligned} & {\begin{matrix} C_6 = & \frac{ (R_1 + 1 + D)^4 - (R_1 + H)^4 }{16} \\ & + C_4 \left[ \frac{(R_1 + 1 + D)^2}{2} \text{ln} (R_1 + 1 + D) - \frac{(R_1 + H)^2}{2} \text{ln} (R_1 + H) + \frac{ (R_1 + H)^2 - (R_1 + 1 + D)^2 }{4} \right] \\ & + C_5 \frac{ (R_1 + 1 + D)^2 - (R_1 + H)^2 }{2}, \end{matrix}} \end{aligned}$$

12 $$\begin{aligned} & {\begin{matrix} C_7 = & \frac{\partial C_4}{\partial Z} \left[ \frac{(R_1 + 1 + D)^2}{2} \text{ln} (R_1 + 1 + D) - \frac{(R_1 + H)^2}{2} \text{ln} (R_1 + H) + \frac{ (R_1 + H)^2 - (R_1 + 1 + D)^2 }{4} \right] \\ & + \frac{\partial C_5}{\partial Z} \frac{ (R_1 + 1 + D)^2 - (R_1 + H)^2 }{2}, \end{matrix}} \end{aligned}$$

13 $$\begin{aligned} & C_4 = \frac{ (R_1 + H)^2 - (R_1 + 1 + D)^2 }{ 4 \, \text{ln} \left( \frac{R_1 + 1 + D}{R_1 + H}\right) }, \end{aligned}$$

14 $$\begin{aligned} & C_5 = - \frac{(R_1 + H)^2}{4} - C_4 \, \text{ln}(R_1 + H). \end{aligned}$$

### Governing equations of the brain parenchyma

We adopt the Biot’s theory [5, 13] to describe the poroelasticity of the parenchyma

15 $$\begin{aligned} & \nabla \cdot \varvec{\sigma } = \alpha \nabla p, \end{aligned}$$

16 $$\begin{aligned} & \alpha \frac{\partial \epsilon _\text{tr}}{\partial t} + \frac{1}{M} \frac{\partial p}{\partial t} = - \nabla \cdot \textbf{v}, \end{aligned}$$

where $\textbf{v} = (v_x,v_z) = - \frac{k}{\eta } \nabla p$ is the Darcy velocity of the ISF flow. Here, we assume that ISF has the same viscosity $\eta$ as CSF, since both fluids share similar properties with water [26, 38]. The tensor $\varvec{\sigma } = \lambda \epsilon _\text{tr} \textbf{I} + 2 \mu \varvec{\epsilon }$ represents the stress in the brain tissue, where $\lambda$ and $\mu$ are Lamé’s first and second parameter, and $\textbf{I}$ is the identity matrix. The strain tensor $\varvec{\epsilon } = (\nabla \mathbf {u_d} + \nabla \mathbf {u_d}^T)/2$ is determined by the deformation field $\mathbf {u_d} = (u_{dx}, u_{dz})$ of the brain tissue. The term $\epsilon _\text{tr} = \text{tr} (\varvec{\epsilon })$ represents the trace of the strain tensor. Since the living brain tissue is incompressible and fully saturated by the ISF, we set the Biot coefficient $\alpha = 1$ and the Biot Modulus (*M*) infinity, making the second term on the left-hand side of Eq. 16 negligible [7, 10, 28, 29]

At the permeable interface with the SAS ($z = 0$), the hydrostatic pressure is set to the SAS pressure and the tissue surface is stress free

17 $$\begin{aligned} p \,\vert _{z=0} = p_\text{SAS} = 0, \quad \varvec{\sigma } \cdot \textbf{n} \,\vert _{z=0} = 0, \end{aligned}$$

where $\textbf{n}$ is the outward unit normal vector at the boundary. At the deep parenchyma ($z = l$), we impose a zero normal derivative for both pressure and the *x*-component of velocity. We assume no displacement of tissue at this boundary, as this region is embedded within the brain parenchyma and is therefore more mechanically constrained compared to the surface boundary ($z = 0$), which is exposed to the SAS. The boundary conditions at $z = l$ are given by

18 $$\begin{aligned} \frac{\partial p}{\partial z} \,\vert _{z=l} = 0, \quad \frac{\partial v_x}{\partial z} \,\vert _{z=l} = 0, \quad \mathbf {u_d} \,\vert _{z=l} = 0. \end{aligned}$$

Within the brain parenchyma domain $\Omega$, the horizontal *x*-direction is defined with its left boundary at the periarterial glial layer (i.e., $x = 0$ in the reference configuration) and its right boundary at the perivenous glial layer (i.e., $x = l_p$ in the reference configuration). These boundaries correspond to the interfaces between the brain parenchyma and the PVSs. We note that in the current (deformed) configuration, these interfaces move in time; for simplicity of notation, we impose $x = 0$ and $x = l_p$ in the reference configuration and account for the displacement through the deformation field $\mathbf {u_d}$. On these interfaces, we enforce continuity of pressure and fluid flux across the glial layers, as well as stress-free conditions on the tissue, matching the parenchyma variables on the glial interfaces with those in the periarterial (subscript $\cdot _A$) and perivenous spaces (subscript $\cdot _V$)

19 $$\begin{aligned} p \,\vert _{x=0}= & p_A (z, t), \quad \varvec{\sigma } \cdot \textbf{n} \,\vert _{x=0} = 0, \quad v_x \,\vert _{x=0} = q_A (z, t), \quad u_{dx} \,\vert _{x=0} = d_A (z, t), \end{aligned}$$

20 $$\begin{aligned} p \,\vert _{x=l_p}= & p_V (z, t), \quad \varvec{\sigma } \cdot \textbf{n} \,\vert _{x=l_p} = 0, \quad v_x \,\vert _{x=l_p} = - q_V (z, t), \quad u_{dx} \,\vert _{x=l_p} = - d_V (z, t). \end{aligned}$$

where $p_A(z,t)$ and $p_V(z,t)$ are leading-order pressure in the PVSs, defining the parenchymal interstitial pressure on the glial interfaces. The fluxes $q_A(z,t)$ and $q_V(z,t)$ represent the cross-glial fluid flux between the PVSs and the parenchyma. The deformations $d_A(z,t)$ and $d_V(z,t)$ are the radial displacements of the glial layers from the PVS model, matching the parenchymal deformation at the glial interfaces. These variables, $p_A$, $p_V$, $q_A$, $q_V$, $d_A$, and $d_V$, are unknowns shared by both PVS and parenchyma models and are solved in a coupled system.

To derive the dimensionless form of the governing equations of parenchyma, the spatial coordinate system is scaled by length *l*, i.e. $x = lX$ and $z = lZ$, which implies that the gradient operator scales as $\nabla = \frac{1}{l} \nabla ^*$, where $\nabla ^*$ is the gradient operator in the dimensionless form. The remaining dimensional variables are scaled as

21 $$\begin{aligned} l_p = l L_p, \quad \mathbf {u_d} = l \mathbf {U_d}, \quad \textbf{v} = lf \textbf{V}, \quad \varvec{\sigma } = \mu \varvec{\sigma ^*}. \end{aligned}$$

Specifically, as the interstitial pressure in the parenchyma is coupled with the PVS pressure via the glial interfaces, both pressures share the same scaling. Thus, the parenchymal interstitial pressure is scaled by the same factor as in the PVS model, i.e., $p = \frac{\eta f}{\varepsilon ^2} P$, where *P* is the dimensionless parenchymal pressure. The dimensionless governing equations become

22 $$\begin{aligned} & \mu ^* \nabla ^* \cdot \varvec{\sigma ^*} = \nabla ^* P, \end{aligned}$$

23 $$\begin{aligned} & \frac{\partial \epsilon _\text{tr}}{\partial T} = - \nabla ^* \cdot \textbf{V}, \end{aligned}$$

24 $$\begin{aligned} & \textbf{V} = - K \nabla ^* P, \end{aligned}$$

where the dimensionless tissue stiffness $\mu ^* = \mu \varepsilon ^2 / (\eta f)$ compares the elastic force to viscous force in brain parenchyma, and the dimensionless tissue permeability $K = k / b^2$ compares the viscous forces between the parenchyma and the PVS. The boundary conditions of dimensionless form read

25 $$\begin{aligned} P \,\vert _{Z=0}= & 0, \quad \varvec{\sigma ^*} \cdot \textbf{n} \,\vert _{Z=0} = 0. \end{aligned}$$

26 $$\begin{aligned} & \frac{\partial P}{\partial Z} \,\vert _{Z=1} = 0, \quad \frac{\partial V_x}{\partial Z} \,\vert _{Z=1} = 0, \quad \mathbf {U_d} \,\vert _{Z=1} = 0. \end{aligned}$$

27 $$\begin{aligned} P \,\vert _{X=0}= & P_{0A}, \quad \varvec{\sigma ^*} \cdot \textbf{n} \,\vert _{X=0} = 0, \quad V_x \,\vert _{X=0} = \varepsilon Q_A, \quad U_{dx} \,\vert _{X=0} = \varepsilon D_A, \end{aligned}$$

28 $$\begin{aligned} P \,\vert _{X=L_p}= & P_{0V}, \quad \varvec{\sigma ^*} \cdot \textbf{n} \,\vert _{X=L_p} = 0, \quad V_x \,\vert _{X=L_p} = - \varepsilon Q_V, \quad U_{dx} \,\vert _{X=L_p} = - \varepsilon D_V. \end{aligned}$$

where $Q_A$ and $Q_V$ are dimensionless fluxes across periarterial and perivenous glial layers, respectively. $D_A$ and $D_V$ are the dimensionless radial deformations of the glial layers on the arterial and venous sides, respectively. The slenderness $\varepsilon$ in Eqs. 27 and 28 originates from the different characteristic length scales chosen for the PVS and parenchyma models. Specifically, the length scale of the fluxes ($Q_A$ and $Q_V$) and deformations ($D_A$ and $D_V$) is set to the width of the PVS (*b*), while the length scale of the parenchymal interstitial velocity $\textbf{V}$ and deformation $\mathbf {U_d}$ is set to *l*.

### Numerical algorithm

We developed an in-house C++ solver to numerically solve the governing Eqs. 9, 22, 23, and 24. Equation 9 of $P_0$ in the PVS is solved along the *z*-direction. The length of PVS *l* is uniformly discretized into $N_z$ points, such that the grid size is $\Delta z = l / (N_z - 1)$. Although the overall PVS model is 2D axisymmetric, Eq. 9 depends only on *z* and time *t*, as the radial (*r*) dependence has been integrated out through the lubrication approximation. As a result, the PVS model can be solved without discretizing *r*-direction. Once the pressure distribution $P_0(Z,T)$ is obtained, it can be used to analytically reconstruct the full 2D velocity field in the PVS [14, 43]. We employ a finite difference method with second-order accuracy to discretize Eq. 9. The parenchyma domain $\Omega$ is discretized on a 2D grid to solve Eqs. 22, 23, and 24. The domain is uniformly segmented into $N_x$ points in *x*-direction and $N_z$ points in *z*-direction, such that the grid size is $\Delta x = l_p / (N_x - 1)$ and $\Delta z = l / (N_z - 1)$. We use finite difference methods with second-order accuracy for spatial discretization.

A numerical stability issue arises in fluid-structure interaction problems involving deformable and permeable glial layers, especially when the coupled systems are solved separately. In prior studies where both deformation and permeability of the glial layer were considered within a single PVS, the cross-glial flux *Q* and glial deformation *D* were expressed explicitly in terms of the PVS pressure $P_0$, allowing the term $(\frac{\partial D}{\partial T} + Q)$ in Eq. 9 to be treated implicitly [14, 43]. In our model, however, the relationships among *D*, *Q*, and $P_0$ are determined not only by the local PVS dynamics but also by the parenchyma model. Furthermore, the dynamics within one PVS can influence the other through their interaction mediated by the parenchyma, which motivates solving the dynamics of both PVSs simultaneously. We therefore developed a semi-implicit algorithm that partially treats the term $(\frac{\partial D}{\partial T} + Q)$ in Eq. 9 implicitly, accounting for system coupling and PVS-parenchyma-PVS interaction.

As we solve the dynamics of the periarterial and perivenous spaces simultaneously, we group their discretized variables into vectors: $\textbf{D} = [D_A,D_V]^T$, $\textbf{Q} = [Q_A,Q_V]^T$, $\mathbf {P_0} = [P_{0A},P_{0V}]^T$, and $\textbf{H} = [H_A,H_V]^T$. Assuming the PVS pressure $\mathbf {P_0}$ is given, we solve Eqs. 22, 23, and 24 for the deformation ($D_A$, $D_V$) and flux ($Q_A$, $Q_V$) at the glial layers. This relationship can be expressed as

29 $$\begin{aligned} \textbf{D} = \mathcal {D}(\mathbf {P_0}, \mathbf {U_d}^\text{prev}), \quad \textbf{Q} = \mathcal {Q}(\mathbf {P_0}, \mathbf {U_d}^\text{prev}), \end{aligned}$$

where the operators $\mathcal {D}$ and $\mathcal {Q}$ represent the process of solving the parenchyma equations to obtain the interstitial pressure *P* and deformation field $\mathbf {U_d}$, given the PVS pressure $\mathbf {P_0}$ and the deformation field from the previous time step $\mathbf {U_d}^\text{prev}$. The glial deformation ($\textbf{D}$) and cross-glial flux ($\textbf{Q}$) are then computed from this solution. Due to the linearity of Eqs. 22, 23, and 24, the mapping operators can be further expressed as

30 $$\begin{aligned} \textbf{D} = \mathcal {M_D}\cdot \mathbf {P_0} + \mathcal {V_D}, \quad \textbf{Q} = \mathcal {M_Q} \cdot \mathbf {P_0} + \mathcal {V_Q}, \end{aligned}$$

where $\mathcal {M_D}$ and $\mathcal {M_Q}$ are matrices representing the contribution of $\mathbf {P_0}$ to $\textbf{D}$ and $\textbf{Q}$, and $\mathcal {V_D}$ and $\mathcal {V_Q}$ are vectors incorporating the effects of variables at previous time step. See the “Derivation of mapping matrices and vectors” section in the Supplementary Information for details on how $\mathcal {M_D}$, $\mathcal {M_Q}$, $\mathcal {V_D}$, and $\mathcal {V_Q}$ are constructed. Using these expressions, we can solve the pressure in the periarterial and perivenous spaces simultaneously and fully couple their interaction through the parenchyma

31 $$\begin{aligned} \mathbf {C_6} \frac{\partial ^2 \mathbf {P_0}}{\partial Z^2} + \mathbf {C_7} \frac{\partial \mathbf {P_0}}{\partial Z} + (R_1 + 1 + \textbf{D}) \left[ \frac{ \mathcal {M_D}}{\text{d}t} + \mathcal {M_Q} \right] \cdot \mathbf {P_0} = \frac{\partial \textbf{H}}{\partial T} (R_1 + \textbf{H}) + (R_1 + 1 + \textbf{D}) \left[ \frac{\textbf{D}}{\text{d}t} - \frac{ \mathcal {V_D}}{\text{d}t} - \mathcal {V_Q} \right] , \end{aligned}$$

where $\mathbf {C_6}$ and $\mathbf {C_7}$ are vectors combining the coefficients $C_6$ and $C_7$ for both periarterial and perivenous spaces.

At each time step, the mapping operators $\mathcal {M_D}$, $\mathcal {M_Q}$, $\mathcal {V_D}$, and $\mathcal {V_Q}$ are first determined based on the variables from previous time step. We then solve Eq. 31 using the current $\textbf{H}$ and $\textbf{D}$ from the previous step. The updated pressure $\mathbf {P_0}$ is used to update $\textbf{D}$, $\textbf{Q}$, as well as the hydrostatic pressure (*P*), deformation ($\mathbf {U_d}$), and velocity ($\textbf{V}$) within the parenchyma. As the simulation starts with an initial guess, we test the convergence of the solution until the glymphatic transport profile over a full oscillation period is stable. The full procedure is summarized in Algorithm 1, and its schematic representation. We perform a grid independence study to ensure that the numerical results are independent of the grid size, as detailed in the Supplementary Information. We confirm that the results converge as the mesh is refined. We choose $N_x = 60$ and $N_z = 150$ in this study, which provides a balance between accuracy and computational efficiency.

**Algorithm 1:**
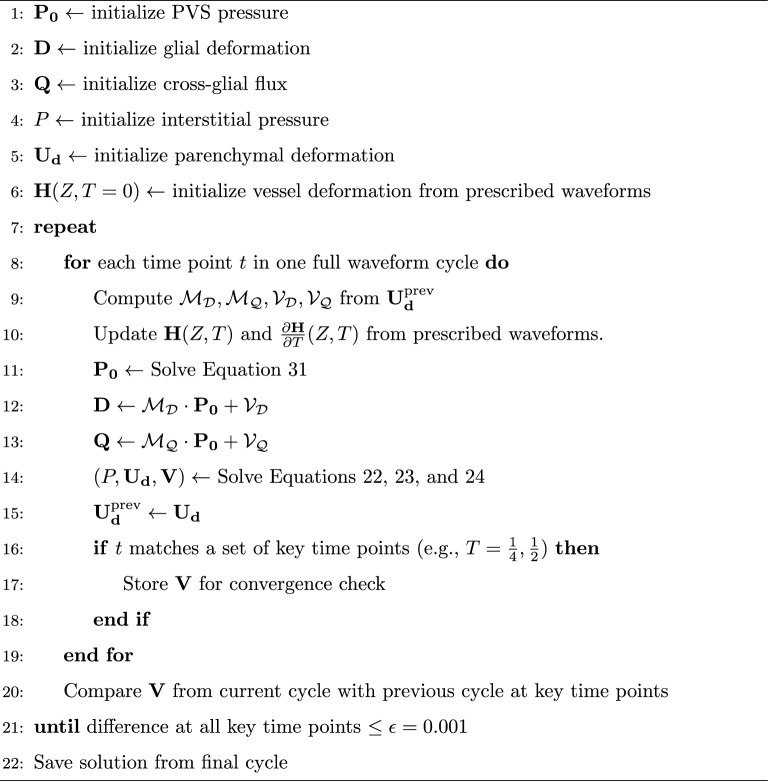
Numerical solution of coupled PVS-parenchyma dynamics

### Parameter selection

As we introduce the model, we summarize here the definitions and values of the parameters used in our simulations. Our model is built in a nondimensional manner, in part to represent the phenomenon across species. To construct a baseline and define physiologically reasonable parameter ranges, we base our choices primarily on mouse anatomy. With limited resources available on the precise phase relationship between arterial and venous deformation, we approximate the phase difference $\psi _{AV} = 0.01\pi$ so that the blood vessel deformation wave propagation through the capillary is on the same scale as that of the artery and vein $2\pi /\Lambda _w = 0.02\pi$. Recognizing uncertainties in this estimation, we perform a parametric study in the Results section, where the phase difference $\psi _{AV}$ is shown to enhance glymphatic transport. In vivo and ex vivo studies have reported *E* ranges from approximately 0.5 to $10 \; \text{kPa}$ [8, 25, 34, 53]. Some computational models have employed higher stiffness values to simulate the deformation of glial layers, up to $1 \; \text{MPa}$ [14, 43]. We select $E = 5 \; \text{kPa}$ for our baseline simulations, as it falls within the reported range of brain tissue stiffness and reflects the soft nature of brain tissue. To explore the influence of tissue stiffness on glymphatic transport, we extend the range of *E* up to $1 \; \text{MPa}$ in parametric study. To represent the nearly incompressible brain tissue, we set the Poisson ratio to $\nu = 0.499$. This value is consistent with experimental measurements reporting high Poisson ratios in brain tissue and avoids the numerical singularity associated with strictly incompressible materials ($\nu = 0.5$) [35]. The dimensionless amplitude of the arterial wall deformation amplitude ($\bar{H}_A$) has been measured to be approximately $0.5 - 2 \%$ [33], and values ranging $\bar{H}_A = 0.5 - 5 \%$ have been employed in previous modeling studies [25, 43]. In the baseline case, we set $\bar{h}_A = 0.5 \; \mathrm {\mu m}$ (corresponding to $\bar{H}_A = 0.05$). We later show in the Discussion (see also Supplementary Information section “Effect of vessel wall deformation amplitude”) that the observed mechanism remains robust when $\bar{H}_A$ is reduced toward the lower physiological range. The deformation amplitude of the venous wall ($\bar{H}_V$) and the ratio $\bar{H}_V/\bar{H}_A$ remain poorly characterized experimentally. The lower pressure in veins, resulting from viscous drag across the capillaries, coupled with the lower stiffness of venous walls compared to arterial walls [50], suggests that venous wall deformation may be smaller or comparable to that of the artery. For the baseline study, we assume the deformation amplitudes of the artery and vein to be of the same order. The frequency and wavelength of the arterial and venous wall motions are kept the same. To assess sensitivity to the venous wall deformation amplitude, we vary $\bar{H}_V$ from 0 to slightly larger than the arterial deformation amplitude to explore its influence on perivenous dynamics and overall glymphatic transport. The value of essential dimensional parameters and their ranges used in our simulation are listed in Table 1, and the dimensionless parameters and their ranges are listed in Table 2.

**Table 1:** Parameters and their ranges used in our simulation

Parameter	Range	Unit	Description	Reference
$\alpha$	1	−	Biot coefficient	
*M*	$\infty$	−	Biot modulus	
*k*	$10^{-14}$ ($10^{-17}$ - $4 \times 10^{-14}$)	$\text{m}^{2}$	hydraulic permeability of tissue	[21]
$\eta$	$10^{-3}$	$\mathrm {Pa \cdot s}$	dynamic viscosity of CSF	[6]
*E*	5 (0.5 - $10^3$)	$\text{kPa}$	Young’s modulus	[8, 14, 25, 34, 43, 53]
$\nu$	0.499	−	Poisson’s ratio	[3, 35]
$\lambda$	$E \nu /[(1+\nu )(1-2\nu )]$	$\text{Pa}$	Lamé’s first parameter	
$\mu$	$E /[2(1+\nu )]$	$\text{Pa}$	Lamé’s second parameter	
*b*	10(1–10)	$\mathrm {\mu m}$	PVS neutral width	[22, 43]
*l*	1000	$\mathrm {\mu m}$	PVS length	[45]
*f*	5 (3–5)	$\text{Hz}$	blood vessel deformation frequency	[14, 18]
$l_p$	250 (175–280)	$\mathrm {\mu m}$	characteristic distance between PVSs	[40]
$r_1$	10	$\mathrm {\mu m}$	blood vessel neutral radius	[43]
$\bar{h}_A$	0.5 (0.05–0.5)	$\mathrm {\mu m}$	amplitude of arterial wall deformation	[33, 43]
$\bar{h}_V$	0.5 (0.05–0.5)	$\mathrm {\mu m}$	amplitude of venous wall deformation	
$\lambda _w$	0.1 (0.004–0.7)	$\text{m}$	wavelength of blood vessel deformation wave	[43]
$\psi _{AV}$	0.01$\pi$ (0–0.2$\pi$)	−	phase difference

**Table 2:** Dimensionless parameters and their ranges used in our simulation

Parameter	Definition	Range	Description
$\varepsilon$	*b*/*l*	0.01	slenderness of PVS
$R_1$	$r_1/l$	1	dimensionless blood vessel neutral radius
$L_p$	$l_p/l$	0.25 (0.175–0.28)	dimensionless characteristic distance between PVSs
$\bar{H}_A$	$\bar{h}_A/b$	0.05 (0.005–0.05)	dimensionless amplitude of arterial wall deformation
$\bar{H}_V$	$\bar{h}_V/b$	0.05 (0.005–0.05)	dimensionless amplitude of venous wall deformation
$\Lambda _w$	$\lambda _w/l$	100 (4–700)	dimensionless blood vessel deformation wavelength
$\mu ^*$	$\mu \varepsilon ^2 / (\eta f)$	33.4 (3.34–6671.1)	dimensionless tissue stiffness
*K*	$k / b^2$	$10^{-4}$ ($10^{-7}$ - $4 \times 10^{-4}$)	dimensionless tissue permeability

## Results

### Perivascular interaction and net directional convection within parenchyma

We first focus on the oscillatory dynamics of tissue deformation and the directional convection driven by the pulsation of blood vessels. Since the wavelength of vessel deformation is much larger than the length of PVS, the blood vessels deform ($H_A$, $H_V$) almost uniformly along the length of the PVS, with a slight tilt superimposed in the deformation wave, as depicted in Fig. 1B, F. The arterial deformation ($H_A$) slightly leads the venous deformation ($H_V$) in phase, as shown in Fig. 1G. This oscillatory vessel deformation modulates the pressure within the PVS, which, in turn, induces oscillatory deformation in the surrounding glial layers ($D_A$, $D_V$). Due to the interaction between the perivascular spaces through the poroelastic parenchyma, the glial deformation is asymmetric, with a bias towards the perivenous side, as depicted in Fig. 1C, E. This asymmetry suggests that the dynamics of PVSs are not individually isolated but instead coupled with each other. Additionally, the periarterial glial layer deforms ($D_A$) slightly earlier than the perivenous glial layer ($D_V$), preserving the phase lead of periarterial dynamics, as shown in Fig. 1G. The time-averaged pressure and velocity across the parenchyma are captured in Fig. 1D, suggesting that the pulsatile deformation of blood vessels generates a net convection across the parenchyma. This convection originates from the periarterial glial layer and flows across the entire parenchyma. In the shallow region of the parenchyma, the convection includes a velocity component parallel to the PVS, driving some fluid directly into the SAS across the surface of the tissue. In the deeper region of parenchyma, the velocity is primarily directed from the periarterial space towards the perivenous space, with the velocity magnitude being smaller compared to the shallow region. We denote averaging over a quantity or domain $\beta$ by the operator $\langle \cdot \rangle _\beta$. The average flow across the domain is computed as $\langle |\langle \textbf{V} \rangle _T |\rangle _\Omega$, where $\langle \textbf{V} \rangle _T (X,Z)$ is the time-averaged velocity at each location (*X*, *Z*), $|\cdot |$ denotes the magnitude, and $\langle \cdot \rangle _\Omega$ represents spatial averaging over the parenchyma domain $\Omega$. The resulting average flow rate across the domain is $\langle |\langle \textbf{V} \rangle _T |\rangle _\Omega = 1.41 \times 10^{-6}$, corresponding to the dimensional value $7.03 \; \mathrm {nm / s}$. To examine the contributors to net directional transport, we conducted a series of controlled simulations that isolate the roles of directional vessel wall wave propagation and the phase difference between arterial and venous wall motion. The results show that both factors contribute to the observed net directional transport, as detailed in the Supplementary Information section “Dissecting contributors to directional glymphatic transport”.

### Averaged artery-to-vein transport by local oscillatory flow

**Fig. 2:**
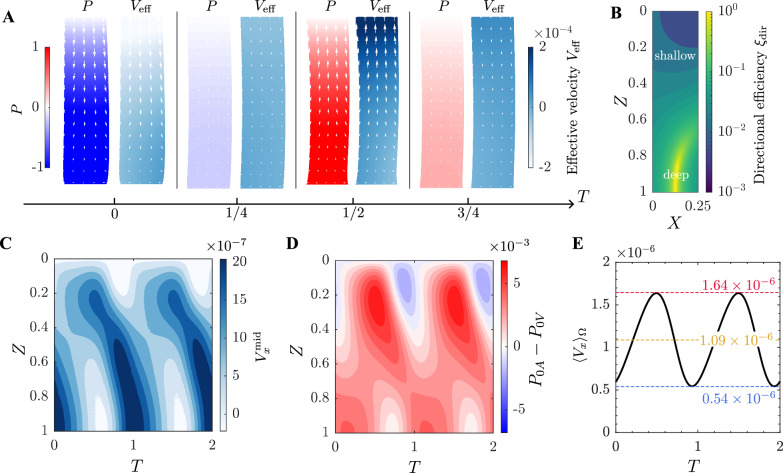
Dynamic pressure and oscillatory convection in the glymphatic system. **A** Spatial and temporal evolution of pressure and effective velocity ($V_\text{eff}$) within the parenchyma at four key time points ($T = 0,\; 1/4,\; 1/2,\; 3/4$). Arrows indicate the direction and magnitude of the velocity fields. **B** Directional efficiency $\xi _\text{dir}$ within the parenchyma. **C** Spatial and temporal variation of artery-to-vein transport velocity on the midplane ($V_x^\text{mid}$). **D** Temporal and spatial evolution of the pressure difference between periarterial and perivenous spaces ($P_{0A} - P_{0V}$). **E** Temporal evolution of spatially averaged artery-to-vein transport velocity across the parenchyma ($\langle V_x \rangle _\Omega$)

**Fig. 3:**
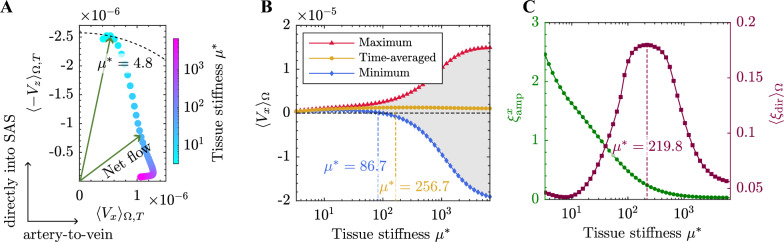
The effect of tissue stiffness on glymphatic transport. (**A**) Variation of the time-averaged velocity components $\langle V_x \rangle _{\Omega , T}$ (artery-to-vein) and $\langle - V_z \rangle _{\Omega , T}$ (directly into the SAS) as a function of tissue stiffness $\mu ^*$. (**B**) Maximum, time-averaged, and minimum values of the velocity component $\langle V_x \rangle _{\Omega }$ as a function of tissue stiffness $\mu ^*$. The shaded region between the maximum (red) and minimum (blue) curves represents the amplitude of oscillation. The orange dashed line marks the tissue stiffness at which the time-averaged flow reaches its maximum. The blue dashed line indicates the critical tissue stiffness where the minimum value crosses zero. (**C**) Amplitude efficiency $\xi ^x_\text{amp}$ (green) and spatially averaged directional efficiency $\langle \xi _\text{dir} \rangle _\Omega$ (purple) as a function of tissue stiffness $\mu ^*$. The purple dashed lines indicates the tissue stiffness values corresponding to the peaks of $\langle \xi _\text{dir} \rangle _\Omega$

Flow within both the PVS and the parenchyma varies over the vasomotion cycle, and the net transport is the result of these dynamic oscillations. In the PVS, the peak velocity at the entrance ($z = 0$) reaches $1.03 \; \mathrm {mm/s}$, which is higher, yet remains within the same order of magnitude as peak values ($\sim 300 \; \mathrm {\mu m/s}$) reported in surface PVSs [26]. The net flow into the periarterial space, however, is only $33.6 \; \mathrm {nm/s}$, balancing with the net flow across the parenchyma under the blind-ended condition imposed in the model. A detailed velocity profile in the PVS is provided in the Supplementary Information section “Flow within the PVS”, along with additional context on its relevance to the experimental findings. In the parenchyma, the velocity field varies in both space and time, alternating between aligning with and diverging from the net directional convection during each oscillation cycle. To capture this dynamic process, we introduce the effective velocity $V_\text{eff} (X,Z,T) = \textbf{V} \cdot \langle \textbf{V} \rangle _T / |\langle \textbf{V} \rangle _T |$ that measures the component of the oscillating velocity field $\textbf{V} (X,Z,T)$ that aligns with the time-averaged directional convection $\langle \textbf{V} \rangle _T (X,Z)$. Unlike $\langle \textbf{V} \rangle _T$, which is a steady, overall measure of flow direction over time, $V_\text{eff}$ varies throughout each oscillation cycle, providing insight into how effectively the instantaneous velocity aligns with the targeted average direction. Figure 3A captures the temporal evolution of pressure *P* and effective velocity $V_\text{eff}$ at four key moments within the oscillation cycle. At times $T = 0$ and $T = 1/2$, corresponding to the maximum rates of vessel contraction and expansion, we observe large magnitudes of both convection velocity and tissue deformation. The pressure variations drive flow primarily parallel to the PVS in the shallow regions of parenchyma, whereas in deeper regions, the velocity components are more perpendicular to the PVS. At $T = 1/4$ and $T = 3/4$, when blood vessels reach their maximal and minimal deformations, the pressure gradient, and thus the convection, are minimal. The spatial variation of $V_\text{eff}$ reveals a contrast between the shallow and deep regions. In the shallow region, $V_\text{eff}$ exhibits notable temporal oscillations, indicating a strong back-and-forth motion superimposed on the overall directional convection. This is reflected in Fig. 2A through the alternating light and dark blue regions over time. In contrast, the deeper regions show relatively uniform coloring and weaker temporal variation in $V_\text{eff}$, suggesting a more consistent flow aligned with net directional convection.

To quantify the efficiency of this transport, we introduce the directional efficiency $\xi _\text{dir}$, defined as the ratio of the net transport velocity ($|\langle \textbf{V} \rangle _T |$) to the total motion in the direction of the net transport over one oscillation period ($\int _{0}^{1} |\textbf{V} \cdot \langle \textbf{V} \rangle _T |\; \text{d}T$). As illustrated in Fig. 2B, the deep parenchyma has higher directional efficiency compared to the shallow region. Notably, in the middle area between the periarterial and perivenous spaces, the directional efficiency approaches 1, indicating an almost unidirectional artery-to-vein transport in this region. For further illustration, we examine the spatial and temporal variation of *x*-direction velocity on midplane ($V_x^\text{mid}$), as shown in Fig. 2C. At each depth within the parenchyma, the flow exhibits an oscillatory pattern where the forward (artery-to-vein) transport predominates over occasional weak backflow. This unidirectional artery-to-vein transport arises from the perivascular interaction. Although the pressure in both periarterial and perivenous spaces oscillate, the pressure in the periarterial space is almost always higher than that in the perivenous space, as illustrated in Fig. 2D. This persistent pressure gradient results in a sustained bulk transport from artery to vein throughout the oscillation period, as depicted in Fig. 2E.

### Impact of brain tissue stiffness on glymphatic transport dynamics

In previous sections, we demonstrated that the interaction between PVSs significantly influences glymphatic transport, mediated through the poroelasticity of the parenchyma tissue. Although some experimental studies suggest that brain tissue stiffens with age and in AD [15, 17, 46], while softening during sleep [15, 16], the overall effect of stiffness on glymphatic clearance remains unclear due to inconsistent findings across different studies. Here, we focus on the impact of brain tissue stiffness on glymphatic transport. Both the direction and magnitude of the glymphatic net flow are affected by tissue stiffness, as shown in Fig. 3A. With softer tissue, the net flow is dominated by its $-z$ component, leading part of the convection in the parenchyma directly into the SAS. As stiffness increases, the net directional flow gradually rotates, aligning more perpendicular to the PVSs. This rotation leads to an increased convection from the periarterial space toward the perivenous space. The magnitude of the directional net transport initially increases, peaking at a tissue stiffness of $\mu ^* = 4.8$ (corresponding to $E = 719.5 \; \text{Pa}$), and then decreases. The peak value $E = 719.5 \; \text{Pa}$ falls within the reported range of brain tissue stiffness ($0.5 - 10 \; \text{kPa}$, corresponding to $\mu ^* = 3.34 - 66.7$), indicating that physiological stiffness levels may support efficient glymphatic transport. We further discuss the implications of this in the Discussion section.

The oscillation pattern of artery-to-vein bulk transport is also affected by tissue stiffness, as illustrated in Fig. 3B. As tissue stiffness increases, the net artery-to-vein transport increases and then decreases, reaching a peak at $\mu ^* = 256.7$ ($E = 38.5 \; \text{kPa}$). This value exceeds the reported range of brain tissue stiffness ($0.5 - 10 \; \text{kPa}$), representing a condition in which the brain may be too stiff to effectively mediate interactions between PVSs. At this high tissue stiffness and beyond (reflecting a lack of perivascular interaction), the oscillation amplitude of $\langle V_x \rangle _\Omega$ becomes much larger than the net flow velocity itself, suggesting a strong back-and-forth bulk transport between periarterial and perivenous spaces with only a small net artery-to-vein flow. In contrast, with softer tissue ($\mu ^* < 86.7$ or $E < 12.9 \; \text{kPa}$), a unidirectional bulk transport from periarterial space to perivenous space is observed. To quantify the persistence of artery-to-vein transport, we introduce the amplitude efficiency $\xi ^x_\text{amp}$, defined as the ratio between the net artery-to-vein flow rate $\langle V_x \rangle _{\Omega , T}$ and its oscillation amplitude. As shown in Fig. 3C, $\xi ^x_\text{amp}$ decreases monotonically as tissue stiffness increases. Although persistent unidirectional artery-to-vein transport is observed with soft tissue, the spatially averaged directional efficiency $\langle \xi _\text{dir} \rangle _\Omega$ across the parenchyma remains relatively low for all $\mu ^*$ within the studied range, with a peak observed at $\mu ^* = 219.8$ ($E = 32.9 \; \text{kPa}$, which exceeds the reported physiological range).

### Phase-delayed venous vasomotion enhances glymphatic transport

**Fig. 4:**
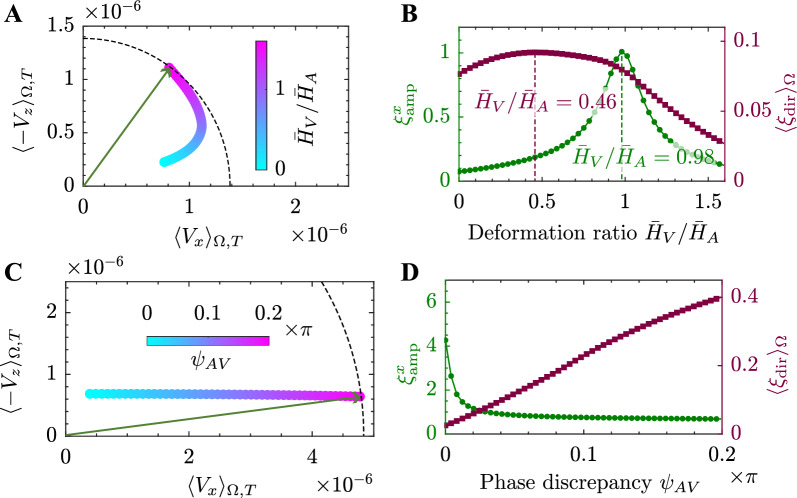
**The effect of venous vasomotion on glymphatic transport.** (**A**) Variation of the time-averaged velocity components $\langle V_x \rangle _{\Omega , T}$ (artery-to-vein) and $\langle - V_z \rangle _{\Omega , T}$ (directly into the SAS) as a function of deformation ratio $\bar{H}_V / \bar{H}_A$. (**B**) Amplitude efficiency $\xi ^x_\text{amp}$ (green) and spatially averaged directional efficiency $\langle \xi _\text{dir} \rangle _\Omega$ (purple) as a function of $\bar{H}_V / \bar{H}_A$. The green and purple dashed lines indicate the peaks of $\xi ^x_\text{amp}$ and $\langle \xi _\text{dir} \rangle _\Omega$, respectively. (**C**) Variation of the $\langle V_x \rangle _{\Omega , T}$ and $\langle - V_z \rangle _{\Omega , T}$ as a function of phase difference $\psi _{AV}$. (**D**) $\xi ^x_\text{amp}$ (green) and $\langle \xi _\text{dir} \rangle _\Omega$ (purple) as a function of $\psi _{AV}$. The green dashed lines indicate the peak of $\xi ^x_\text{amp}$

Because both arteries and veins deform in response to oscillatory blood pressure waves, glymphatic transport is driven not only by arterial vasomotion but also by venous vasomotion, with the perivascular interaction modulating the overall effect. Here we investigate the role of venous vasomotion, specifically the deformation ratio between vein and artery ($\bar{H}_V / \bar{H}_A$) and their phase difference ($\psi _{AV}$), on the glymphatic transport. As illustrated in Fig. 4(A), net glymphatic transport exist even without venous vasomotion ($\bar{H}_V / \bar{H}_A = 0$). As the deformation of the vein increases, the net flow rotates to align more with the PVS, and its magnitude increases monotonically, suggesting that venous vasomotion enhances convective transport. Figure 4B shows that the amplitude efficiency of artery-to-vein bulk transport ($\xi ^x_\text{amp}$) reaches a peak when the vein’s deformation amplitude is slightly less than that of the artery ($\bar{H}_V / \bar{H}_A = 0.98$). As the vein deformation amplitude decreases from this point, the spatially averaged directional efficiency across the parenchyma $\langle \xi _\text{dir} \rangle _\Omega$ exhibits a nonmonotonic behavior, peaking when the vein amplitude is about half that of the artery ($\bar{H}_V / \bar{H}_A = 0.46$).

Due to blood flow through capillaries between arteries and veins, a small phase difference ($\psi _{AV}$) exists between their deformations. When this phase difference is neglected ($\psi _{AV} = 0$), a small net flow still occurs, primarily aligning with the PVS, facilitating fluid exchange between the tissue and SAS, as shown in Fig. 4(C). As the phase difference increases, the *z*-component of the net flow remains relatively constant, while the *x*-component (artery-to-vein) increases monotonically, leading to stronger glymphatic transport. With larger phase discrepancies, the amplitude efficiency $\xi ^x_\text{amp}$ decreases monotonically to a plateau value of 0.61. Its high value at small phase discrepancy indicates a high persistence of artery-to-vein bulk transport when the arterial and venous vasomotion are closely in phase. Conversely, the spatially averaged directional efficiency $\langle \xi _\text{dir} \rangle _\Omega$ increases monotonically with larger phase discrepancies, indicating enhanced overall directional efficiency of glymphatic transport.

## Discussion

In this study, we developed a multiphysics model to investigate the dynamics of glymphatic transport within the brain. Our model captures the interactions between periarterial spaces, perivenous spaces, and the brain parenchyma. Our results indicate that the directional glymphatic transport is modulated by the interaction between PVSs, driving a unidirectional bulk transport from the periarterial space to the perivenous space. To our knowledge, this is the first work that captures the nonmonotonic effect of brain tissue stiffness on glymphatic transport. This finding may help understand the implications of prior experimental observations in aging and AD, where both increased and decreased brain stiffness have been reported. Our results suggest that deviations from an optimal stiffness, in either direction, may impair glymphatic function. Moreover, our results provide insights into how perivenous dynamics influence the glymphatic transport within the brain.

One of the key findings of our model is the significant role of perivascular interaction in establishing net directional convection within the brain parenchyma. Throughout an oscillation period, the deformation of glial layers presents an asymmetric pattern, indicating the coupling of their dynamics. Even though the pressure in both periarterial and perivenous spaces oscillates between positive and negative values, the perivascular interaction results in the pressure in the periarterial space being consistently higher than that in the perivenous space. As a result, even though local backflow exists, the bulk transport across parenchyma is always from artery to vein. The net glymphatic convection originates from the periarterial space sweeping through the parenchyma, with part of the flow entering the perivenous space and part directly flowing into the SAS. We also found that the same mechanism holds across a range of deformation amplitudes, including values down to $50 \; \text{nm}$, with a corresponding decrease in net transport magnitude (see Supplementary Information section “Effect of vessel wall deformation amplitude”). The time-averaged pressure in both periarterial and perivenous spaces is higher than the pressure in the SAS, suggesting that the perivenous space is not merely functioning as a passive sink but actively participates in transport dynamics.

To estimate the significance of glymphatic convection in the waste clearance, we evaluate the Péclet number in the brain parenchyma $\text{Pe} = l_p \langle |\langle \textbf{v} \rangle _T |\rangle _\Omega / (\phi D^*)$, where $\phi = 0.2$ is the porosity of the parenchyma, and $D^* = 10^{-12} - 10^{-11}\; \mathrm {m^2/s}$ is the diffusivity. Based on our results, the average glymphatic convection rate across the domain $\langle |\langle \textbf{v} \rangle _T |\rangle _\Omega$ is approximately $10 \; \mathrm {nm/s}$. This leads to a Péclet number in the parenchyma ranging from 1.2 to 12.5, indicating that glymphatic convection is comparable to or dominant over diffusion in brain waste clearance.

Our investigation into the impact of brain tissue stiffness suggests that both the net glymphatic transport and its efficiency are sensitive to tissue stiffness. An extremely stiff brain, characterized by the absence of perivascular interactions, results in poor convective transport efficiency, which aligns with predictions in the literature [2, 14, 42]. Notably, our findings demonstrate that within the experimentally measured range of brain stiffness ($0.5 - 10 \; \text{kPa}$), glymphatic transport presents a stronger net flow comparing to scenarios without perivascular interactions, further highlighting the crucial role of perivascular interaction. We observed a nonmonotonic trend in both net glymphatic transport and its efficiency. Specifically, as brain stiffness increases beyond the peak transport magnitude, the net transport decreases, while the artery-to-vein transport is enhanced, accompanied by increased directional efficiency. These results are especially intriguing given the varying effects of brain tissue stiffness reported in experimental studies. Our model offers two potential explanations: (1) Physiological brain tissue stiffness may have a value that maximizes glymphatic transport. Deviations from this optimal stiffness-whether increase or decrease-could compromise glymphatic clearance ability. (2) Increased brain tissue stiffness may lead to a decreased net flow rate. However, the enhancement in directional efficiency might result in more effective waste clearance, considering that the transportability of metabolic waste could be influenced by the local convection rate. This suggests that the overall effectiveness of metabolic waste clearance might be an intricate balance between flow rate and directional efficiency, both of which are influenced by tissue stiffness. Note that brain tissue porosity has also been suggested to enhance glymphatic transport during sleep. Our model captures this trend, showing a monotonic increase in glymphatic transport with porosity, in agreement with experimental observations (see Supplementary Information section “Effect of tissue porosity on the glymphatic transport”). This suggests that tissue porosity, as an additional tissue property alongside stiffness, may also influence glymphatic transport.

In exploring how venous vasomotion affect the glymphatic dynamics, we found that a phase-delayed venous vasomotion enhances the magnitude of glymphatic transport. This suggests that not only the amplitude but also the timing of venous pulsations relative to arterial pulsations is critical for optimal glymphatic function. The amplitude and timing of venous pulsation are closely related to various physiological conditions, including age-related vascular changes, exercise-induced cardiac changes, disease-related blood pressure changes, etc. To further understand how this mechanism functions in vivo, further experimental validation is needed, particularly in measuring venous vasomotion and its impact on transport mechanism in the brain.

Our study has some limitations. Firstly, the simplified geometries do not capture the full complexity of the brain’s vasculature and tissue architecture. Future models could consider more detailed anatomical structures, including the branching of capillaries and the inhomogeneous and heterogeneous tissue properties, to more accurately represent the in vivo condition. The model also adopts idealized values for local geometric parameters, such as vessel and PVS radii. To maintain symmetry, we assume that arteries and veins share the same radii and the width of surrounding PVSs. This simplification allows the periarterial and perivenous spaces to be treated in a comparable framework, enabling us to isolate and study the effects of parameters such as wall deformation amplitude and phase difference. Mechanistically, the width of the PVS may influence the degree of coupling between vessel wall motion and the glial layer deformation. While our model uses symmetric geometries for clarity and control, we acknowledge that asymmetries between arterial and venous geometry could influence perivascular interactions and glymphatic transport. These effects could be explored in future studies. Additionally, in deriving the model, we assume that the outlets of PVSs are blind-ended. Some anatomical studies, however, suggest that these spaces may be interconnected through pericapillary spaces. Due to significant uncertainties in the anatomical existence and the dynamic properties of these pericapillary spaces, we chose to adopt the blind-end assumption in this work. Nonetheless, the potential existence of pericapillary spaces is worth exploring in future studies to better understand their implications for glymphatic dynamics. Furthermore, we model the PVSs as open fluid-filled channels, using the Navier-Stokes equations to describe the fluid flow. This assumption reflects current understanding of the surface PVSs, while the structure of penetrating PVSs remains poorly characterized. If these spaces are instead porous, a different modeling framework would be required. Given the current lack of consensus on the structure of penetrating PVSs, we acknowledge this as an important open question for future model development. Our model focuses on the mechanical interactions and does not account for biochemical factors that may affect glymphatic transport, such as the role of AQP-4 channels on the glial layers [47]. Integrating these aspects could provide a more comprehensive understanding of glymphatic functions and the relative importance of each contributing mechanism. For our input parameters, we have benefited from examining the effects of individual factors separately (such as phase difference) when exploring the parameter space. Future efforts could further correlate these inputs with physiological conditions. For instance, simulating the effects of exercise could allow multiple parameters to vary simultaneously, helping validate our model and expand its relevance to medical and pharmaceutical applications. Finally, another area for future research is to consider the nonlinearity of mass transport and viscoelastic properties of brain tissue. While we used Biot’s theory to model the poroelastic behavior and used the net glymphatic transport to represent the ability of metabolic waste clearance, incorporating more complex mechanical behavior could reveal additional information on how tissue mechanics affect glymphatic transport.

## Supplementary Information

Below is the link to the electronic supplementary material.

Supplementary file (PDF 1484 KB)

## Data Availability

All data generated or analyzed during this study are included in this published article.
