# Supplementary material for: Perivascular interactions and tissue properties modulate directional glymphatic transport in the brain
Source: Fluids Barriers CNS. 2025 Jun 23;22:63. doi: 10.1186/s12987-025-00668-3 (PMC12183829; doi:10.1186/s12987-025-00668-3)
Supplement: Supplementary file 1 — Supplementary file (PDF 1484 KB) [file 12987_2025_668_MOESM1_ESM.pdf]

Supplementary Information

Derivation of mapping matrices and vectors

Here we describe how the matrices $\mathcal{M}_\mathcal{D}$ and $\mathcal{M}_\mathcal{Q}$, as well as the vectors $\mathcal{V}_\mathcal{D}$ and $\mathcal{V}_\mathcal{Q}$, are constructed in the numerical scheme. Assuming the PVS pressure $\mathbf{P_0}$ is given, the numerical solver of the parenchyma model can be written as

$$\mathcal{A}_1 \cdot [P, U_{dx}, U_{dy}]^T = [\mathbf{P_0}, U_{dx}^{\text{prev}}, U_{dy}^{\text{prev}}, ...]^T, \tag{1}$$

where $\mathcal{A}_1$ is the coefficient matrix constructed by discretizing the governing equations of the parenchyma. The vector $[P, U_{dx}, U_{dy}]^T$ includes the unknowns, including the interstitial pressure P and the components of the deformation field U_{dx} and U_{dy}. The right-hand side includes the PVS pressure $\mathbf{P_0}$, the deformation field at the previous time step (U_{dx}^{prev}, U_{dy}^{prev}), and other terms resulting from the boundary condition settings (e.g., the stress-free boundary condition on the tissue). The solved system is given by

$$[P, U_{dx}, U_{dy}]^T = \text{inv}(\mathcal{A}_1) \cdot [\mathbf{P_0}, U_{dx}^{\text{prev}}, U_{dy}^{\text{prev}}, ...]^T, \tag{2}$$

where $\text{inv}(\mathcal{A}_1)$ is the inverse of the coefficient matrix $\mathcal{A}_1$. The solution of this system can be used to compute the glial deformation $\mathbf{D}$ and the cross-glial flux $\mathbf{Q}$

$$\mathbf{D} = \mathcal{A}_2 \cdot [P, U_{dx}, U_{dy}]^T, \tag{3}$$

$$\mathbf{Q} = \mathcal{A}_3 \cdot [P, U_{dx}, U_{dy}]^T, \tag{4}$$

where the matrices $\mathcal{A}_2$ and $\mathcal{A}_3$ represent numerical operators applied on the solution of the parenchyma model. These linear operators can be combined to express the dependence of $\mathbf{D}$ and $\mathbf{Q}$ on the PVS pressure $\mathbf{P_0}$

$$\mathbf{D} = \mathcal{A}_4 \cdot [\mathbf{P_0}, U_{dx}^{\text{prev}}, U_{dy}^{\text{prev}}, ...]^T, \tag{5}$$

$$\mathbf{Q} = \mathcal{A}_5 \cdot [\mathbf{P_0}, U_{dx}^{\text{prev}}, U_{dy}^{\text{prev}}, ...]^T, \tag{6}$$

where $\mathcal{A}_4 = \mathcal{A}_2 \cdot \text{inv}(\mathcal{A}_1)$, and $\mathcal{A}_5 = \mathcal{A}_3 \cdot \text{inv}(\mathcal{A}_1)$ are known matrices that can be constructed from the numerical scheme.

We now extract the indices of the matrices $\mathcal{A}_4$ and $\mathcal{A}_5$ that correspond to the contribution of $\mathbf{P_0}$ to the value of $\mathbf{D}$ and $\mathbf{Q}$, forming the matrices $\mathcal{M}_\mathcal{D}$ and $\mathcal{M}_\mathcal{Q}$, respectively. The remaining contributions from $[U_{dx}^{\text{prev}}, U_{dy}^{\text{prev}}, ...]^T$ are collected into the vectors $\mathcal{V}_\mathcal{D}$ and $\mathcal{V}_\mathcal{Q}$, thus leading to expressions

$$\mathbf{D} = \mathcal{M}_\mathcal{D} \cdot \mathbf{P_0} + \mathcal{V}_\mathcal{D}, \quad \mathbf{Q} = \mathcal{M}_\mathcal{Q} \cdot \mathbf{P_0} + \mathcal{V}_\mathcal{Q}, \tag{7}$$

where the matrices $\mathcal{M}_\mathcal{D}$ and $\mathcal{M}_\mathcal{Q}$, and the vectors $\mathcal{V}_\mathcal{D}$ and $\mathcal{V}_\mathcal{Q}$, are known and can be used to formalize the semi-implicit solver for $\mathbf{P_0}$.

Grid independence study

We perform a grid independence study to ensure that the numerical results are independent of the grid size. As shown in Figure S1, we compute the average flow rate across the domain ($\langle|\langle \mathbf{V}\rangle_T|\rangle_\Omega$) as a function of the number of grids ($N_x \times N_z$) in the mesh. The results demonstrate that the average flow rate approaches to plateau as the number of grids increases, indicating convergence and grid independence. Balancing accuracy and computational efficiency, we choose $N_x = 60$ and $N_z = 150$ for all the simulations in this study. At this resolution, the average flow rate across the domain ($\langle|\langle \mathbf{V}\rangle_T|\rangle_\Omega$) deviates by less than 0.5% from the converged value.

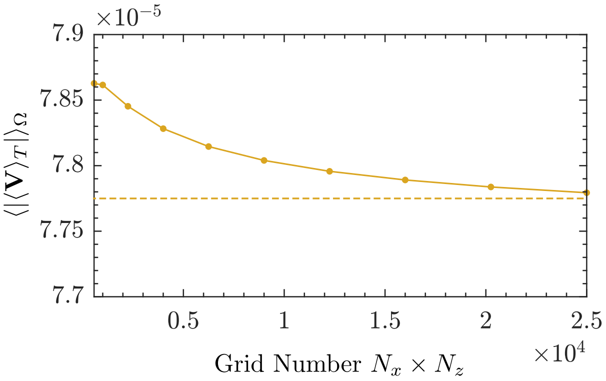

Figure S1: **Grid independence study.** The average flow rate across the domain ($\langle|\langle \mathbf{V}\rangle_T|\rangle_\Omega$) as a function of the number of grids ($N_x \times N_z$) in the mesh.

Dissecting contributors to directional glymphatic transport

To identify the physical contributors responsible for the net glymphatic transport in the baseline model, we designed a series of controlled simulations that isolate two contributing factors: (1) the directional wave propagation in the arterial and venous walls, which can generate a net pressure difference between the periarterial and perivenous spaces via peristaltic pumping when combined with the blind-ended geometry; and (2) the phase difference between the arterial and venous wall deformations, which can lead to net glymphatic transport through perivascular interaction. To dissect the contributions of these two factors, we compared the baseline condition to three modified cases: (1) baseline, propagative wave ($\Lambda_w = 100$) and phase difference ($\psi_{AV} = 0.01\pi$); (2) minimum phase difference ($\psi_{AV} = 0$), while preserving wave propagation ($\Lambda_w = 100$); (3) no propagative deformation ($\Lambda_w \to \infty$), so each vessel wall deforms as a bulk along its length l, isolating the effect of phase difference $\psi_{AV} = 0.01\pi$; and (4) no perivascular interaction ($\mu^* \to \infty$), modeling a rigid brain in which coupling between PVSs is effectively removed.

Figure S2 summarizes the net glymphatic transport and the temporal evolution of artery-to-vein transport for each case. In the (1) baseline case, net glymphatic transport from periarterial space to perivenous space is observed, with some fluid directly entering the SAS. The bulk artery-to-vein flow ($\langle V_x\rangle_\Omega$) is unidirectional.

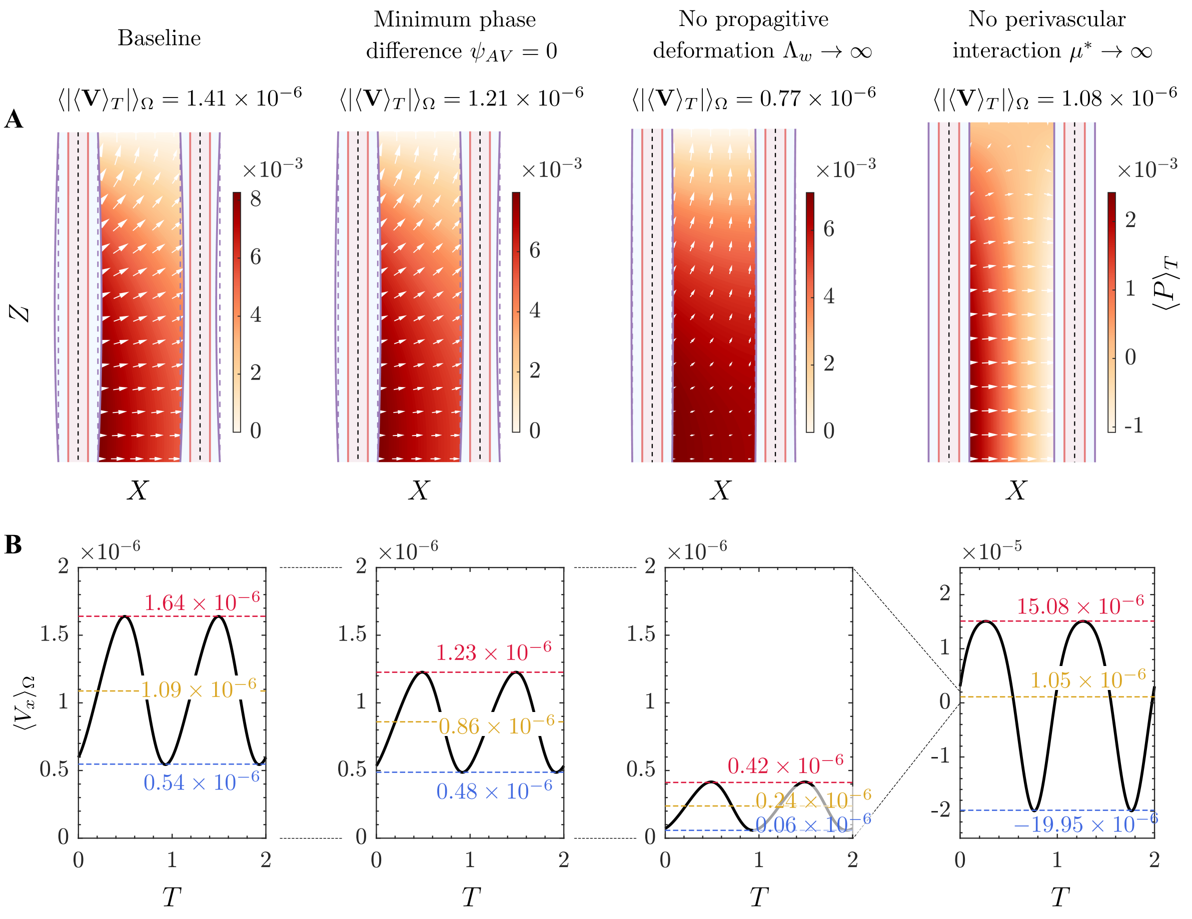

Figure S2: **Glymphatic transport under controlled simulation settings.** (**A**) Time-averaged pressure and velocity fields within the parenchyma under each setting. The averaged flow across the domain is shown above each plot. (**B**) Temporal evolution of spatially averaged artery-to-vein transport velocity across the parenchyma ($\langle V_x\rangle_\Omega$) under each setting.

The observed flow is driven by both the directional wave propagation and the phase difference. In case (2), where the phase difference ψ_{AV} is set to zero, net transport is still present but reduced in magnitude. This confirms that phase difference is not strictly required to generate net flow, though it enhances transport. Notably, even at $\psi_{AV} = 0$, arterial and venous wall deformations are not fully synchronized due to the directional propagation: the deformation at $z = l$ on the venous wall is in phase with the deformation at $z = 0$ on the arterial wall when $\psi_{AV} = 0$. As a result, the spatially averaged artery deformation $\langle H_A \rangle_Z$ remains slightly ahead of $\langle H_V \rangle_Z$ in time. Case (3) isolates the contribution of phase difference by enforcing each vessel wall to deform synchronously along its length ($\Lambda_w \to \infty$). A net flow is still observed, primarily parallel to the PVS. While the artery-to-vein component is weaker, it remains unidirectional, confirming that even a small phase difference can drive net flow through perivascular interaction when each vessel wall deforms synchronously along its length. As the phase difference becomes larger, the artery-to-vein component increases significantly, as demonstrated in more detail in the main text section "Phase-delayed venous vasomotion enhances glymphatic transport". Case (4) suppresses perivascular interaction by modeling a rigid brain. Under this configuration, the periarterial and perivenous spaces are nearly uncoupled. Net flow is observed from artery to vein, now driven purely by pressure differences resulting from directional wave propagation. However, the magnitude is lower than in the baseline case, and the artery-to-vein velocity shows strong oscillatory behavior. Without coupling, the pressure in the periarterial space is no longer persistently higher than that in the perivenous space.

These results show that both directional wave propagation and phase difference contribute to the net glymphatic transport observed in the baseline case, mediated by the poroelastic brain tissue. When the phase difference is small, directional wave propagation contributes to most of the artery-to-vein transport. At larger phase differences, phase difference becomes the primary driver of the net glymphatic transport, a trend explored in more detail in the main text section "Phase-delayed venous vasomotion enhances glymphatic transport".

Flow within the PVS

Based on lubrication theory (Romanò et al., 2020; Gan et al., 2023), the leading-order terms of the dimensionless velocity field within the PVS (U_{z0}, U_{r0}) can be determined from the leading-order pressure (P_0) as follows

$$U_{z0} = \frac{R^2}{4}\frac{\partial P_0}{\partial Z} + C_1(Z,T)\ln R + C_2(Z,T), \tag{8}$$

$$U_{r0} = -\frac{R^3}{16}\frac{\partial^2 P_0}{\partial Z^2} - \frac{\partial C_1(Z,T)}{\partial Z}\left(\frac{R}{2}\ln R - \frac{R}{4}\right) - \frac{R}{2}\frac{\partial C_2(Z,T)}{\partial Z} + \frac{C_3(Z,T)}{R}. \tag{9}$$

Here, the coefficients $C_1(Z,T)$, $C_2(Z,T)$, and $C_3(Z,T)$ are determined by

$$C_1 = C_4\frac{\partial P_0}{\partial Z}, \quad C_4 = \frac{(R_1+H)^2 - (R_1+1+D)^2}{4\ln\left(\frac{R_1+1+D}{R_1+H}\right)}, \tag{10}$$

$$C_2 = C_5 \frac{\partial P_0}{\partial Z}, \quad C_5 = -\frac{(R_1+H)^2}{4} - C_4 \ln(R_1+H). \tag{11}$$

$$C_3 = (R_1+H)\left\{\frac{\partial H}{\partial T} + \frac{(R_1+H)^3}{16}\frac{\partial^2 P_0}{\partial Z^2} + \frac{\partial C_1}{\partial Z}\left[\frac{R_1+H}{2}\ln(R_1+H) - \frac{R_1+H}{4}\right] + \frac{R_1+H}{2}\frac{\partial C_2}{\partial Z}\right\} \tag{12}$$

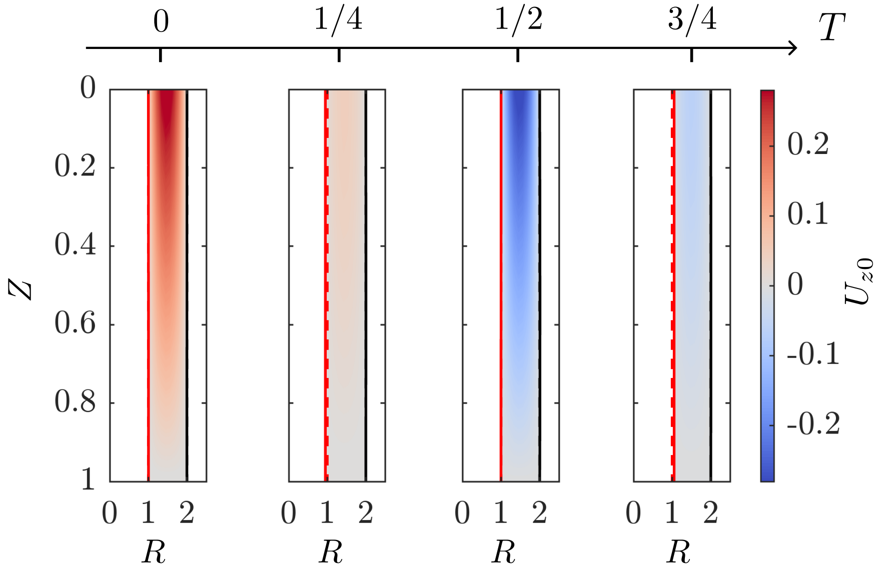

Figure S3: **Oscillatory flow in the PVS.** Spatial and temporal evolution of leading-order dimensionless axial velocity (U_{z0}) within the periarterial space at four key time points ($T = 0,\ 1/4,\ 1/2,\ 3/4$).

Figure S3 shows the spatiotemporal evolution of the leading-order axial velocity U_{z0} within the periarterial space at four key moments ($T = 0,\ 1/4,\ 1/2,\ 3/4$) over the vasomotion cycle. The flow in the periarterial space is predominantly oscillatory, with a peak velocity of 1.03 mm/s at the entrance of the periarterial space ($z = 0$), which is higher, yet remains within the same order of magnitude as the peak instantaneous velocities ($\sim 300\ \mu$m/s) reported in surface PVS experiments (Kelley & Thomas, 2023). In contrast to the relatively high oscillatory speed, the net velocity at the entrance is 33.6 nm/s. This small value results from the imposed blind-ended boundary condition, under which net flow into the PVS balances with the net flow into the parenchyma. Compared to existing experimental findings, our simulations do not reproduce the sustained directional transport reported in some surface PVS studies (Mestre et al., 2018). However, the small net flow we observe aligns with the tracer experiments showing limited penetration into the penetrating periarterial space (Iliff et al., 2012). It remains an open question whether flow in the penetrating PVS is primarily oscillatory or directional, and experimental results remain inconclusive on this point. Our model suggests a PVS flow dominated by oscillation, with net flow balanced with the fluid exchange through the parenchyma. Alternative anatomical assumptions, such as direct connections between periarterial and perivenous spaces via pericapillary spaces, may lead to more pronounced directional flow. These possibilities are further discussed in the Discussion section. Other computational studies have also reported oscillatory flow in the PVS driven by arterial vasomotion, while a systemic pressure gradient along the PVS can generate net flow velocities that quantitatively comparable to experimental findings (Daversin-Catty et al., 2020).

Effect of vessel wall deformation amplitude

To examine whether the observed transport persists across the physiological range of vessel deformation amplitude, we varied the amplitude from 50 nm to 0.5 μm (corresponding to $\bar{H}_A = 0.005 - 0.05$) , while maintaining a constant deformation ratio ($\bar{H}_V/\bar{H}_A = 1$).

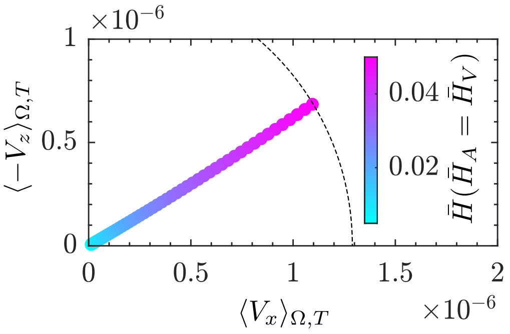

Figure S4: **The effect of vessel deformation amplitude on glymphatic transport.** Variation of the time-averaged velocity components $\langle V_x \rangle_{\Omega,T}$ (artery-to-vein) and $\langle -V_z \rangle_{\Omega,T}$ (directly into the SAS) as a function of vessel deformation amplitude $\bar{H}(\bar{H}_A = \bar{H}_V)$.

Figure S4 shows that the net glymphatic flow increases monotonically with vessel deformation amplitude. The direction of the net glymphatic flow remains consistent across the amplitudes, while only the magnitude varies. Even at the lowest amplitude tested (50 nm), the model preserves the same transport mechanism observed in the baseline case, with a smaller magnitude of the transport.

Effect of tissue porosity on the glymphatic transport

In the foundational glymphatic study by Xie et al. (Xie et al., 2013), the porosity of the mouse brain was reported to increase from approximately 0.14 during wakefulness to 0.23 during sleep. This increase was associated with an enhanced glymphatic clearance. The authors proposed that a smaller interstitial space during wakefulness increases hydraulic resistance, thereby impeding interstitial fluid transport. To incorporate this effect into our model, we scale the permeability k with porosity ϕ by

$$\frac{k}{k^{\text{base}}} = \frac{\phi^3(1-\phi^{\text{base}})^2}{\phi^{\text{base}^3}(1-\phi)^2}, \tag{13}$$

where the baseline values are $\phi^{\text{base}} = 0.2$ and $k^{\text{base}} = 1.0 \times 10^{-14}$ m^2 (Carman, 1937).

Figure S5 shows that higher porosity leads to a monotonic increase in glymphatic transport. This model prediction is consistent with the experimental observation that glymphatic clearance is enhanced during sleep, when porosity increases. These results suggest that tissue porosity plays a role in modulating glymphatic transport, serving as an additional tissue property alongside brain stiffness.

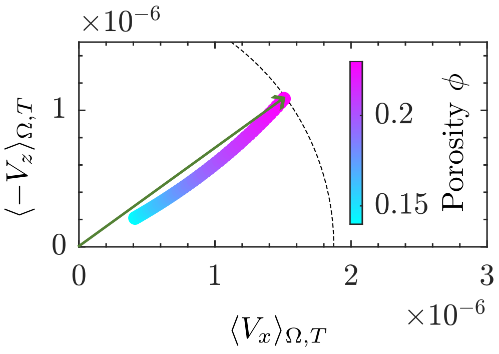

Figure S5: **The effect of tissue porosity on glymphatic transport.** Variation of the time-averaged velocity components $\langle V_x \rangle_{\Omega,T}$ (artery-to-vein) and $\langle -V_z \rangle_{\Omega,T}$ (directly into the SAS) as a function of tissue porosity ϕ.

References

Carman, P. C. (1937). Fluid flow through granular beds. *Trans. Inst. Chem. Eng. London*, *15*, 150–156.

Daversin-Catty, C., Vinje, V., Mardal, K.-A., & Rognes, M. E. (2020). The mechanisms behind perivascular fluid flow. *Plos one*, *15*, e0244442.

Gan, Y., Holstein-Rønsbo, S., Nedergaard, M., Boster, K. A., Thomas, J. H., & Kelley, D. H. (2023). Perivascular pumping of cerebrospinal fluid in the brain with a valve mechanism. *Journal of the Royal Society Interface*, *20*, 20230288.

Iliff, J. J., Wang, M., Liao, Y., Plogg, B. A., Peng, W., Gundersen, G. A., Benveniste, H., Vates, G. E., Deane, R., Goldman, S. A. et al. (2012). A paravascular pathway facilitates csf flow through the brain parenchyma and the clearance of interstitial solutes, including amyloid β. *Science translational medicine*, *4*, 147ra111–147ra111.

Kelley, D. H., & Thomas, J. H. (2023). Cerebrospinal fluid flow. *Annual Review of Fluid Mechanics*, *55*, 237–264.

Mestre, H., Tithof, J., Du, T., Song, W., Peng, W., Sweeney, A. M., Olveda, G., Thomas, J. H., Nedergaard, M., & Kelley, D. H. (2018). Flow of cerebrospinal fluid is driven by arterial pulsations and is reduced in hypertension. *Nature communications*, *9*, 4878.

Romanò, F., Suresh, V., Galie, P. A., & Grotberg, J. B. (2020). Peristaltic flow in the glymphatic system. *Scientific reports*, *10*, 21065.

Xie, L., Kang, H., Xu, Q., Chen, M. J., Liao, Y., Thiyagarajan, M., O'Donnell, J., Christensen, D. J., Nicholson, C., Iliff, J. J. et al. (2013). Sleep drives metabolite clearance from the adult brain. *science*, *342*, 373–377.
